# The impact of educational intervention on enhancing the health literacy among middle-aged individuals at risk for cardiovascular diseases: a randomized cotrolled trial

**DOI:** 10.1186/s12889-025-23516-3

**Published:** 2025-07-03

**Authors:** Seyedeh Fatemeh Azizi Sani Dopolani, Hassan Shahbazi, Masoud Lotfizadeh, Shohreh Naderimagham

**Affiliations:** 1 Department of Health Education and Promotion, Faculty of Medical Sciences, Tarbiat Modares University, Tehran, Iran; 2 Department of Community Health, Faculty of Health, Shahrekord University of Medical Sciences, Shahrekord, Iran; 3 Elderly Health Research Center, Endocrinology and Metabolism Population Sciences Institute, Tehran University of Medical Sciences, Tehran, 1411713137 Iran

**Keywords:** Educational intervention, Health literacy, Cardiovascular diseases

## Abstract

**Background:**

Cardiovascular diseases are the main cause of mortality worldwide. Health literacyplays an important role in reducing the risk of these diseases. This study was conducted to evaluate The Impact of Educational Intervention on Enhancing the Health Literacy among Middle-Aged Individuals at Risk for Cardiovascular Diseases.

**Methods:**

This randomized controlled trial was conducted in 2024 in Shahrekord city, Iran. Initially, a suitable instrument was developed, then, appropriate educational content was designed. In the third phase, the educational intervention was implemented with the participation of 50 individuals in the intervention group and 50 in the control group. A pre-test was administered simultaneously in both groups, followed by a post-test immediately after the intervention and another three months later. Data were analyzed using SPSS 27 software and non-parametric statistical tests including the Mann-Whitney U test, Friedman test, and Wilcoxon test.

**Results:**

The mean score of health literacy and its dimensions showed no significant difference between the intervention and control groups before the educational intervention (*p* > 0.05 for all cases). However, these scores increased significantly in the intervention group compared to the control group both immediately after and three months post-education (*p* < 0.01 for all cases).

**Conclusion:**

The findings indicate the effectiveness of the educational intervention in improving health literacy and its dimensions among individuals at risk of cardiovascular diseases. These results emphasize the importance of incorporating structured educational programs in the national health system.

**Trial registration:**

Iranian Registry of Clinical Trials, IRCT 20240124060797N1. Registered on 15 July 2024, https://irct.behdasht.gov.ir/trial/75187.

## Introduction

The heart, blood vessels, and blood form the components of the human cardiovascular system [1]. Cardiovascular diseases (CVDs) include ischemic heart disease, heart failure, stroke, peripheral arterial disease, and several other cardiovascular conditions [2]. These diseases impact patients’ health-related quality of life and a substantial economic and public health burden on families and healthcare systems [3]. complications of cardiovascular diseases include high healthcare costs, and physical disability [1]. In recent years, CVDs have led to a significant decline in quality of life and life expectancy worldwide [4]. According to the World Health Organization (WHO), are the leading cause of mortality worldwide, leading for 17.9 million deaths each year [5]. Moreover, it is projected that by 2030, this number will rise to 23 million globally [6].

The prevalence and mortality rates of CVDs vary across countries and regions, making a growing concern in many developing countries [7]. In the United States, CVDs are the primary cause of death among adults [8]. Similarly, in Asia, these diseases accounted for nearly 35% of all deaths in 2019 [9]. As the most populous continent, Asia bears a significant burden of CVDs [10]. Iran is among the countries with the highest prevalence of CVDs, with over 9,000 cases per 100,000 individuals [11]. According to a study by Saki et al., based on data of the Prospective Epidmiological Studies of the Iranian Adult and Neonates (PERSIAN), one million disability-adjusted life years (DALYs) are attributed to these diseases [12]. The American Heart Association identifies eight essential factors for cardiovascular health, including a healthy diet, physical activity, avoidance of nicotine exposure, blood pressure control, blood sugar and lipid regulation, and sleep health [13]. Age is a key factor in decline of cardiovascular function, and the risk of developing CVDs increases with the age [14]. Approximately 42% of CVD-related deaths occur in individuals aged 30–69 years [15]. As highlighted by Rethemiotaki, CVDs are primarily observed in older adults and middle-aged individuals [16]. Identifying individuals at risk for cardiovascular diseases and ensuring they receive appropriate treatment is critical for preventing premature mortality [17].

Health literacy promotion interventions are essential tools for primary and secondary prevention of non-communicable diseases [18], and Education is a crucial factor in achieving adequate health literacy [19]. According to the WHO, health education is a process that includes planned educational and communicational opportunities designed to enhance health-related knowledge, skills and life skills for individuals and communities [20]. Overall, health literacy skills play crucial role in health-related decision-making, as they empower individuals with the knowledge and competencies needed to access, understand, and evaluate health information and services, enabling them to use these resources effectively to improve their own health and that of those around them [19]. In chronic diseases, health literacy interventions encompass various approachessuch as counseling, education, self-management techniques, and other methods aimed at improving individuals’ knowledge, attitudes, skills, and behaviors [21]. Adequate health literacy is a prerequisite for adopting healthy behaviors. Low health literacy is associated with increased hospitalization rates, poor self-management, low participation in preventive activities, and higher mortality rates [22]. Individuals with low health literacy face significant challenges in lifestyle management, chronic disease planning, making informed decisions, and recognizing when to seek healthcare services [23]. Among individuals with cardiovascular diseases, low health literacy is linked to adverse outcomes, such as increased mortality, reduced quality of life, and higher rates of hospital readmission [24]. The prevalence of low health literacy in Iran has been reported as 56% [25]. Similarly, a study by Haghdoost et al. indicated inadequate health literacy levels among Iranian adults [26]. Additionally, a study conducted by Reisi et al. in Shahrekord revealed poor health literacy among adults in this city, emphasizing the need for educational interventions to improve health literacy and health-promoting behaviors [27]. Considering the high prevalence of cardiovascular diseases and the inadequate level of health literacy reported in the world, in Iran, and in the city of Shahrekord, with emphasis on the role of education on health literacy, this study was designed and conducted to assess The Impact of Educational Intervention on Enhancing the Health Literacy among Middle-Aged Individuals at Risk for Cardiovascular Diseases.

## Methods

### Study design and participants

The study, conducted in Shahrekord, Iran, in 2024, was an interventional randomized controlled trial (RCT) registered in the Iranian Registry of Clinical Trials, IRCT 20240124060797N1. This study was conducted in accordance with the CONSORT 2010 guidelines.

The study sample consisted of 100 individuals at risk for CVDs, selected using a cluster random sampling method. Initially, one health center from each of the three municipal districts of Shahrekord was randomly selected for the intervention group and another center was randomly selected for the control group using a random number table, resulting in three centers per group. The sample size was calculated using the Pocock formula, with a 95% confidence level and 90% test power, based on a similar previous study [28]. Taking into account a 20% attrition rate, The final sample included 50 individuals in the intervention group and 50 in the control group, who were randomly selected using a random number table. Participants were selected based on the inclusion criteria through the Integrated health system (SIB system), which is available at the health center of each district. Among the middle-aged individuals (30–59 years old) covered by these centers, those at risk of developing cardiovascular diseases were selected for participation. This included individuals with a family history of cardiovascular diseases, substance or alcohol use, diabetes, hypertension, overweight, or obesity. Individuals previously diagnosed with cardiovascular diseases were excluded from the study. The random allocation sequence was generated by the study statistician using a random number table. Enrollment and assignment of participants to the intervention and control groups were carried out by a master’s student in health education and health promotion.

### Study instruments

The data collection instrument for this study consisted of two sections: demographic information and health literacy assessment questions. The health literacy questionnaire used in this study was a Persian language instrument utilized in the study by Saeedi et al. [29]. Since this questionnaire had been previously used with a different target group to assess health literacy related to iron-deficiency anemia, it was re-evaluated for validity and reliability. A panel of eight experts in health education, cardiovascular diseases, and nursing assessed the content validity of the questionnaire.

The Content Validity Ratio (CVR) was reported as greater than 0.75, and the Content Validity Index (CVI) was greater than 0.79. To evaluate reliability, Cronbach’s alpha coefficient was calculated using a sample of 30 individuals representative of the target population, yielding a coefficient greater than 0.7 in all domains.

Regarding demographic variables, the questionnaire contained 13 questions, including information on age, gender, weight, height, education level, occupation, income satisfaction, family history of hypertension and cardiovascular diseases, personal history of hypertension and diabetes, and other relevant factors. The health literacy questionnaire consisted of 19 questions assessing health literacy regarding cardiovascular diseases, with a total score range of 19–95.

The questionnaire included 3 questions for the domains of access skills, communication skills, and media literacy, with a score range of 3–15; 4 questions for the decision-making skills domain, with a score range of 4–20; and 6 questions for the self-management skills domain, with a score range of 6–30. All questions were rated on a 5-point Likert scale, ranging from “never” (score of 1), “rarely” (score of 2), “sometimes” (score of 3), “often” (score of 4), and “always” (score of 5).

### Educational content

Based on pre-test results (Table 1) and the percentage of scores obtained, educational priorities were identified. Educational content was then developed using credible sources, including the WHO, the Centers for Disease Control and Prevention (CDC), the Ministry of Health and Medical Education (MOHME) of Iran, Tehran Heart Center, and findings from previous similar studies.

### Educational intervention

The educational intervention consisted of a structured four-session program conducted over a one-month period at designated health centers, as shown in Table 2. At the beginning of the study, the objectives were clearly explained to all participants, who then signed consent forms including a commitment to refrain from any interaction or exchange of information between groups. To reinforce this commitment, periodic reminders were provided throughout the study. Considering that the selected health centers for the intervention and control groups were located in geographically distinct regions, there was no possibility for participants in the control group to access the intervention sessions or engage in any form of information exchange with members of the intervention group. To further reduce the risk of contamination, neutral group identifiers (Group A/B) were employed, and intervention sessions were scheduled at separate times to avoid any direct contact. Educational materials were securely managed and remained inaccessible to the control group. Finally, anonymous post-study surveys confirmed participants’ adherence to these protocols, thereby ensuring the integrity of the study. Accordingly, participants in the intervention group received the structured educational program, while those in the control group did not receive any educational intervention. Participants were recruited in early May 2024, and the educational intervention was conducted throughout May 2024. Both the intervention and control groups completed the questionnaires at three time points: before the intervention (pre-test), immediately after the intervention (post-test), and three months post-intervention (follow-up). No participants in either group were lost to follow-up or excluded after randomization. To enhance and maintain participant engagement throughout the study, several strategies were employed. At the beginning of the study, the objectives and potential benefits were clearly explained to participants, fostering a sense of value and motivation. The educational sessions were scheduled flexibly to align with participants’ availability. These sessions were designed to be interactive and relevant to participants’ daily lives, which contributed to maintaining active participation and interest. Additionally, regular follow-up calls and reminder messages were made to ensure consistent attendance. Participants were respectfully engaged and their contributions were acknowledged throughout the intervention, fostering a supportive atmosphere that promoted sustained participation.

**Table 1:** Results from the pre-test

Variable	Mean ± SD	Min-Max Score	Percentage of score obtained	Score Level	Priority in Content Design
Self-management skills	13.38 ± 3.53	6–30	44.6%	Medium	1
Decision-making skills	10 ± 2.28	4–20	50.0%	Medium	2
Media literacy skills	8.27 ± 1.66	3–15	55.13%	Medium	3
Communication skills	9.56 ± 1.72	3–15	63.73%	Medium	4
Access skills	9.8 ± 1.4	3–15	65.33%	Medium	5

Note: A percentage score below 33%indicates a poor score level, between 33% and 66% indicates an average score level, and above 66% indicates a good score level

**Table 2:** Action plan

Session	Session Title	Learning Domain	Method	Educatonal Materials	Time & Location	Instructor
1	Communication skills & Access skills	Cognitive, Affective, Psychomotor	Lecture, Practical Training	Pamphlet, PowerPoint, Computer-based system	60 min, Healthcare centers	master’s student in health education and health promotion, noncommunicable disease specialist
2	Media literacy skills & Self-management skills	Cognitive, Affective	Group discussion, Practical Training, Lecture	PowerPoint, Video	60 min, Healthcare centers	master’s student in health education and health promotion
3	Decision-making skills	Cognitive, Affective	Lecture, Group discussion	Educational video	60 min, Healthcare centers	master’s student in health education and health promotion, Mental health expert
4	Self-management skills	Cognitive, Affective	Group discussion, Lecture	PowerPoint, Educational video	60 min, Healthcare centers	master’s student in health education and health promotion, Mental health expert

### Statistical analysis

The collected data were entered into SPSS 27 statistical software. The Kolmogorov-Smirnov test indicated that the data had a non-normal distribution. Therefore, data was analyzed using descriptive statistical methods, including mean, frequency, and relative frequency, as well as non-parametric statistical tests, such as Mann-Whitney U test, Friedman test, and Wilcoxon test. Considering that none of the participants were excluded from the study, the analyses were conducted based on the originally assigned groups.

## Results

The Chi-square test results (Table 3) indicated that there were no statistically significant differences in demographic variables between the intervention and control groups before the intervention (*P* > 0.05 in all cases). This finding confirmed that the study conditions were suitable for conducting the intervention with minimal confounding variables. The mean age of the participants was 42.46 ± 7.68 years in the intervention group and 42.50 ± 7.17 years in the control group.

**Table 3:** Comparison of demographic variables between the intervention and control groups

Variable	Category	Intervention Group (*n* = 50)	Control Group (*n* = 50)	*P*-value
Age (years)	30–40	18 (36.0%)	24 (48.0%)	0.440
	40–50	18 (36.0%)	16 (32.0%)	
	50–59	14 (28.0%)	10 (20.0%)	
Gender	Female	36 (72.0%)	33 (66.0%)	0.517
	Male	14 (28.0%)	17 (34.0%)	
Occupation	Homemaker	27 (54.0%)	19 (38.0%)	0.152
	Government employee	8 (16.0%)	12 (24.0%)	
	Self-employed	10 (20.0%)	17 (34.0%)	
	Retired	5 (10.0%)	2 (4.0%)	
Body Mass Index (BMI)	Underweight	0 (0.0%)	0 (0.0%)	0.069
	Normal	10 (20.0%)	17 (34.0%)	
	Overweight	20 (40.0%)	23 (46.0%)	
	Obese	20 (40.0%)	10 (20.0%)	
Education **Level**	Illiterate	1 (2.0%)	0 (0.0%)	0.097
	Below diploma	16 (32.0%)	6 (12.0%)	
	Diploma	15 (30.0%)	17 (34.0%)	
	Bachelor’s degree	14 (28.0%)	20 (40.0%)	
	Master’s degree	3 (6.0%)	7 (14.0%)	
	Doctorate	1 (2.0%)	0 (0.0%)	
Income satisfaction	Yes	3 (6.0%)	6 (12.0%)	0.452
	No	28 (56.0%)	23 (46.0%)	
	Somewhat	19 (38.0%)	21 (42.0%)	
Family History of Cardiovascular Disease	Yes	44 (88.0%)	36 (72.0%)	0.113
	No	5 (10.0%)	13 (26.0%)	
	Idon’t know	1 (2.0%)	1 (2.0%)	

According to Table 4, regarding intergroup differences, the nonparametric Mann-Whitney test showed that the difference in the mean score of access to health information and services skills between the intervention and control groups was not statistically significant before the educational intervention (*P* = 0.718). However, this difference became significant immediately and three months after the educational intervention (*P* < 0.001).

In terms of intragroup differences, the nonparametric Friedman test indicated that the difference in the mean score of access to health information and services skills in the intervention group before, immediately after, and three months after the educational intervention was statistically significant (*P* < 0.001), whereas in the control group, no significant difference was observed before, immediately after, and three months after the intervention (*P* = 0.203).

Regarding the mean score of communication skills, the nonparametric Mann-Whitney test showed that there was no significant difference between the intervention and control groups before the educational intervention (*P* = 0.151). However, this difference became significant immediately and three months after the intervention (*P* < 0.001).

In terms of intragroup differences, the nonparametric Friedman test indicated that in the intervention group, the difference in the mean score of communication skills before, immediately after, and three months after the educational intervention was statistically significant (*P* < 0.001). In contrast, in the control group, the difference in the mean score of communication skills before, immediately after, and three months after the intervention was not statistically significant (*P* = 0.225).

Based on the results of the nonparametric Mann-Whitney test, the mean score of media literacy skills between the intervention and control groups was not significantly different before the educational intervention (*P* = 0.250). However, this difference became significant immediately and three months after the intervention (*P* < 0.001).

In terms of intragroup differences, the nonparametric Friedman test indicated that in the intervention group, the difference in the mean score of media literacy skills before, immediately after, and three months after the educational intervention was statistically significant (*P* < 0.001). Additionally, in the control group, the difference in the mean score of media literacy skills before, immediately after, and three months after the intervention was also statistically significant (*P* = 0.009).

Furthermore, the mean score of decision-making skills between the intervention and control groups, according to the nonparametric Mann-Whitney test, was not significantly different before the educational intervention (*P* = 0.382). However, this difference became significant immediately and three months after the intervention (*P* < 0.001). In terms of intragroup differences, the nonparametric Friedman test indicated that in the intervention group, the difference in the mean score of decision-making skills before, immediately after, and three months after the educational intervention was statistically significant (*P* < 0.001). Additionally, in the control group, the difference in the mean score of decision-making skills before, immediately after, and three months after the intervention was also statistically significant (*P* = 0.023).

The nonparametric Mann-Whitney test, in examining the mean score of self-management skills, showed that before the educational intervention, there was no significant difference between the intervention and control groups (*P* = 0.251). However, this difference became significant immediately and three months after the intervention (*P* < 0.001). In terms of intragroup differences, the nonparametric Friedman test indicated that in the intervention group, the difference in the mean score of self-management skills before, immediately after, and three months after the educational intervention was statistically significant (*P* < 0.001), whereas in the control group, the difference in the mean score of self-management skills before, immediately after, and three months after the intervention was not statistically significant (*P* = 0.255).

The total health literacy mean score between the intervention and control groups, according to the nonparametric Mann-Whitney test, was not significantly different before the educational intervention (*P* = 0.140). However, this difference became significant immediately and three months after the intervention (*P* < 0.001). In terms of intragroup differences, the nonparametric Friedman test indicated that in the intervention group, the difference in the total health literacy mean score before, immediately after, and three months after the educational intervention was statistically significant (*P* < 0.001). Additionally, in the control group, the difference in the total health literacy mean score before, immediately after, and three months after the inte rvention was also statistically significant (*P* = 0.040).

**Table 4:** Comparison of average scores of health literacy dimensions

Variable	Pre-test	Immediately after intervention	3 months after the intervention	Test result
	Mean ± SD	Mean ± SD	Mean ± SD	
**access to health information and services**				
Intervention	9.74 ± 1.51	12.46 ± 1.36	12.34 ± 1.25	*p* < 0.001
Control	9.86 ± 1.29	10.16 ± 1.66	9.64 ± 1.44	*P* = 0.203
Test result	*P* = 0.718	*p* < 0.001	*p* < 0.001	
**communication skills**				
Intervention	9.26 ± 1.56	12.18 ± 2.05	11.98 ± 1.94	*p* < 0.001
Control	9.86 ± 1.84	10.04 ± 1.89	10.20 ± 1.81	*P* = 0.225
Test result	*P* = 0.151	*p* < 0.001	*p* < 0.001	
**media literacy skills**				
Intervention	8.40 ± 1.75	10.82 ± 2.57	10.48 ± 2.42	*p* < 0.001
Control	8.14 ± 1.55	8.64 ± 1.87	8.74 ± 2.19	*p* = 0.009
Test result	*P* = 0.250	*p* < 0.001	*p* < 0.001	
**decision-making skills**				
Intervention	9.76 ± 2.25	15.50 ± 3.10	15.06 ± 3.10	*p* < 0.001
Control	10.24 ± 2.31	10.46 ± 2.32	10.72 ± 2.86	*P* = 0.023
Test result	*P* = 0.382	*P* < 0.001	*P* < 0.001	
**self-management skills**				
Intervention	12.96 ± 3.10	21.08 ± 5.20	20.64 ± 5.11	*P* < 0.001
Control	13.80 ± 3.90	13.70 ± 3.24	3.76 ± 3.71	*P* = 0.255
Test result	*P* = 0.251	*P* < 0.001	*P* < 0.001	
**total health literacy**				
Intervention	50.12 ± 5.59	72.04 ± 10.09	70.50 ± 10.02	*P* < 0.001
Control	51.90 ± 5.83	53.00 ± 5.37	53.06 ± 6.82	*P* = 0.040
Test result	*P* = 0.140	*P* < 0.001	*P* < 0.001	

## Discussion

The aim of the present study was to determine The Impact of Educational Intervention on Enhancing the Health Literacy among Middle-Aged Individuals at Risk for Cardiovascular Diseases in Shahrekord city, Iran. The findings showed that, for the first dimension of health literacy accessing health information and services the difference in the mean score of access skills between the intervention and control groups was not statistically significant before the educational intervention (*P* = 0.718). This difference became significant immediately after and three months following the intervention (*P* < 0.001). Health education programs and interventions have been shown to significantly improve individuals’ awareness and access to healthcare services [30]. Almeida et al. also reported that education can have a significant impact on the accessability and utilization of healthcare services [31]. In addition, the results of a study conducted by Blaga et al., which focused on improving access to health information in rural settings, were consistent with the findings of the present study. Their study showed that individuals in the intervention group were more engaged in seeking health information and had higher self-efficacy in reading and understanding health-related content [32].

Communication skills serve as an essential medium for doctor-patient interaction, facilitating accurate diagnosis and effective treatment [33]. Good communication skills can lead to improved health outcomes, reduced healthcare costs, and increased patient satisfaction and adherence to medical treatments [34]. According to the present study, the difference in the mean score of communication skills between the two groups was not statistically significant before the educational intervention (*P* = 0.151). The difference became significant immediately after and three months following the intervention (*P* < 0.001). These results are consistent with those of Gutierrez et al., confirming the effectiveness of educational interventions in improving communication skills [35]. A study conducted by Khodadadi et al. also demonstrated the effectiveness of an educational intervention in improving communication skills among nurses in Tabriz, Iran [36].

The results of the present study also indicated that, in terms of intergroup differences, the mean score of media literacy skills did not significantly differ between the two groups before the educational intervention (*P* = 0.250). However, this difference became significant immediately after and three months following the intervention (*P* < 0.001). In today’s world, the media has a central role as educators, especially in the field of health, and have a significant impact on public health [37]. A study by Jeong et al. demonstrated promising results regarding the role of media literacy education in improving health outcomes across various domains [38]. Similarly, Jonesns Jung et al. showed that educational interventions help audiences protect themselves from any harmful effects of misleading information [39]. Based on these findings and similar research, educational interventions appear to be effective in improving media literacy. It is worth noting that in the present study, while the the mean score of media literacy was significantly different before, immediately after, and three months post-intervention in the intervention group, the control group also showed a significant increase However, this increase was minimal compared to the intervention group, which may indicate that media health literacy among the people of Shahrekord is gradually improving.

According to the findings of the present study, the difference in the mean score of decision-making skills between the two groups was not statistically significant before the educational intervention (*P* = 0.382), whereas this difference became significant immediately after and three months following the intervention (*P* < 0.001). A study conducted by Mohebbi et al. demonstrated that health education interventions can effectively enhance decision-making skills and improve cardiovascular health in middle-aged individuals [40]. The findings of this study align with those of Altan et al. [41].

Regarding within-group differences, the mean score of decision-making skills before, immediately after, and three months post-intervention was statistically significant in the intervention group (*P* < 0.001). A significant difference was also observed in the control group before, immediately after, and three months post-intervention (*P* = 0.023); however, this change was minimal compared to the intervention group. As previously mentioned, media literacy skills in the control group suggesting that this increase have contributed to improve their decision-making skills as well.

The results of this study also showed that the mean score of self-management skills between the intervention and control groups did not differ significantly before the educational intervention (*P* = 0.251). However, this difference became significant immediately after and three months following the intervention (*P* < 0.001). A study by Lorig et al. demonstrated that self-management education interventions can significantly improve self-management skills and self-efficacy in managing chronic conditions [42]. Similarly, Okafor et al. confirmed the effectiveness of educational interventions in improving self-management practices among individuals with type 2 diabetes [43].

This study had no significant limitations affecting the validity or generalizability of the findings.

The results of this research indicated that the mean total health literacy score between the two groups did not show a statistically significant difference before the educational intervention (*P* = 0.140). However, this difference became significant immediately after and three months following the intervention (*P* < 0.001). Regarding within-group differences, the mean total health literacy score was statistically significant in the intervention group before, immediately after, and three months post-intervention (*P* < 0.001). Similarly, a meta-analysis conducted by Tavakoly Sany et al. also revealed that health literacy interventions improve health literacy status, self-efficacy, and health-promoting behaviors [44]. These findings are in line with studies conducted by Li et al. [45], confirming the effectiveness of educational interventions in improving health literacy, and by Albus et al. who highlighted the effectiveness of certain interventions in improving health literacy for the prevention of cardiovascular diseases [46].

Additionally, the mean total health literacy score in the control group also showed a statistically significant increase before, immediately after, and three months post-intervention (*P* = 0.040), indicating an improvement in health literacy level in the control group over time. The growing interest in health literacy in recent years, and numerous interventions aimed at enhancing it, has contributed to a steady improvement in health literacy compared to the past. As observed in this study, an increase in media literacy and decision-making skills in the control group also contributed to the overall improvement of health literacy. However, the extent of these changes was significantly greater in the intervention group compared to the control group. Some studies conducted in China indicate that health literacy has improved over time. Over the past decades, significant progress has been made in enhancing various aspects of health literacy among Chinese residents, and it is predicted that this upward trend will continue until 2027 [47, 48]. Furthermore, research conducted by Kucera et al. reported a 12% reduction in individuals with limited health literacy from 2015 to 2020 [49].

## Conclusion

The findings of this study indicate the effectiveness of the designed educational intervention in significantly improving health literacy scores and its dimensions in the context of cardiovascular diseases preventions. Enhancing health literacy among individuals at risk of cardiovascular diseases, particularly middle-aged adults who constitute a significant proportion of cardiovascular disease cases, is a crucial necessity in the country’s healthcare system. This improvement is expected to lead to a substantial reduction in the incidence and mortality rates associated with cardiovascular diseases, not only by strengthening the foundation of healthy families but also by ensuring better health outcomes for the elderly in the future.

This study also demonstrated the positive impact of the educational intervention on improving various dimensions of health literacy among middle-aged individuals at risk of cardiovascular diseases. Our educational content had a significant and positive impact on enhancing communication skills, decision-making abilities, media literacy, access to healthcare services, and self-management skills. Additionally, a slight increase in media literacy and decision-making skills was observed in the control group, which may be attributed to the growing public interest in media literacy and its influenc on health-related decision-making. Furthermore, the healthcare system is actively progressing toward improving health literacy and its components. As mentioned previous studies, public interest in health literacy has been increasing. This one-month educational intervention, conducted in collaboration with Shahrekord University of Medical Sciences, played a vital role in enhancing health literacy and improving health-related behaviors to prevent cardiovascular diseases.

## Data Availability

The data utilized for analysis are available from the corresponding author upon reasonable request.

## Abbreviations

CVDs: Cardiovascular diseases
WHO: World Health Organization
DALYs: disability-adjusted life years
RCT: randomized controlled trial
CVR: Content Validity Ratio
CVI: Content Validity Index
CDC: Centers for Disease Control and Prevention
MOHME: Ministry of Health and Me dical Education
BMI: Body Mass Index
